# Editorial for *Brain Sciences* Special Issue: “Neurogenetic Disorders across Human Life: From Infancy to Adulthood”

**DOI:** 10.3390/brainsci14020111

**Published:** 2024-01-23

**Authors:** Paulo Ribeiro Nóbrega, Pedro Braga-Neto

**Affiliations:** 1 Division of Neurology, Walter Cantidio University Hospital, Federal University of Ceara, Fortaleza 60430-372, CE, Brazil; pbraganeto@ufc.br; 2 Campus Parque Ecológico, Centro Universitário Christus, Fortaleza 60160-230, CE, Brazil; 3 Center of Health Sciences, State University of Ceará, Fortaleza 60714-903, CE, Brazil

This Special Issue assembles papers that highlight different types of neurogenetic disorders that occur throughout human life, from childhood to adulthood, focusing on their natural history, epidemiology, diagnosis, and treatment approaches. Consequently, the manuscripts in this issue focus on rare presentations, expanded phenotypes or the particularities of treatments for neurogenetic diseases. Partially reversible leukoencephalopathy is an intriguing phenotype of variable and sometimes unclear etiology. Barcelos and colleagues report a series of six unrelated patients with subacute regression of developmental milestones and partially reversible leukoencephalopathy associated with *RNASEH2B* pathogenic variants, expanding the spectrum of Aicardi–Goutières syndrome [1]. Latin American and Indian patients are underrepresented in most multicentric studies on genetic disorders, and these populations have a high index of consanguinity [2] with new pathogenic variants and phenotype expansions, as described in recent papers [3,4]. Barcelos and colleagues present a case of Bardet–Biedl Syndrome with congenital hypothyroidism and hearing loss due to compound heterozygous variants in *BBS6*, which is causative of Bardet–Biedl; a homozygous pathogenic variant in the stereocilin (*STRC*) gene associated with deafness; and a homozygous variant in the dual oxidase 2 (*DUOX2*) gene associated with congenital hypothyroidism [1]. Treatable genetic disorders have a significant impact on patient wellbeing, and Ribeiro and colleagues review the treatment approach for cerebrotendinous xanthomatosis, a multisystemic disease with variable neurologic involvement [5], focusing on lipid abnormalities [6]. Regarding potentially treatable disorders, Duchenne muscular dystrophy is the most common neuromuscular disease in humans, and some causative pathogenic variants offer the possibility of targeted treatment. Braga and colleagues describe a large single-center cohort of *DMD* patients with a higher frequency of treatment-amenable variants [7]. Finally, some conditions bridge the gap between autoimmune and genetic disease in both children and adults. Moraes and colleagues discuss autoinflammatory diseases from a neurologist´s perspective, providing interesting insights for clinicians [8]. A comprehensive understanding of neurogenetic diseases from infancy to adulthood may improve diagnostic procedures and provide insights into unmet therapeutic needs [9].
